# Inertial Measurement Unit Based Hip Flexion Strength-Power Test for Sprinters

**DOI:** 10.3389/fspor.2020.571523

**Published:** 2020-10-30

**Authors:** Ryu Nagahara, Munenori Murata

**Affiliations:** National Institute of Fitness and Sports in Kanoya, Kanoya, Japan

**Keywords:** running, acceleration, IMU, fitness test, torque

## Abstract

This study aimed to examine whether a recently developed inertial measurement unit (IMU)-based hip flexion strength-power test could be an indicator of sprint performance, step length (SL) and frequency (SF) during sprinting using sprinters. Sixteen well-trained male sprinters performed 60-m sprints and an IMU-based hip flexion test which consisted of five serial hip flexion-extension motions for each leg with three different conditions (unweighted, 0.75 or 1.5 kg ankle weighted). Running speed, SL and SF from the start to the 50-m mark were measured using a long force platform system. The hip flexion strength-power test variables were collected using one IMU attached to the lateral thigh. The right hip flexion positive work in the 1.5 kg weighted condition was positively correlated with running speed from the 9th−12th to 21st−22nd step sections (*r* = 0.588–0.761) and with SF at the 17th−20th step section (*r* = 0.526). The right hip flexion positive mean power in the 1.5 kg weighted condition was positively correlated with running speed from the 13th−16th to 21st−22nd step section (*r* = 0.547–0.638) and with SF from the 13th−16th to 21st−22nd step section (*r* = 0.501–0.553). The current results demonstrate that, among well-trained male sprinters, hip flexion positive work and mean power measured using IMU-based strength-power test in the 1.5 kg weighted right leg condition can be a determinant of better sprint performance through higher SF during the later acceleration section approaching maximal speed.

## Introduction

Fitness tests are useful for evaluating training induced changes in physical status of athletes. Moreover, to perform such tests is advantageous for weekly or monthly monitoring purposes. The leg and its joints extension strength-power capabilities have been evaluated on the field using several vertical jumps (Bosco et al., 1983; Cronin and Hansen, 2005; Smirniotou et al., 2008; Nagahara et al., 2014a) and hip thrust (Loturco et al., 2018). In contrast, hip or knee joint flexion strength-power capabilities have mainly been evaluated in laboratory settings typically using an isokinetic dynamometer (Farrar and Thorland, 1987; Alexander, 1989; Copaver et al., 2012).

Nagahara et al. (2017) found that the work and mean power produced by hip flexion during sprinting was associated with increases in running speed during sprint acceleration, indicating that the hip flexion strength-power capabilities are important for accomplishing better sprint performance. Therefore, assessing hip flexion strength-power capabilities can be helpful for sprinters and their coaches. Copaver et al. (2012) found that the hip flexion mean power measured using an isokinetic dynamometer was correlated with 50-m sprint time in physically active males. However, no such relationship was found in sprinters (Alexander, 1989). This absence of association in sprinters is likely because the isokinetic dynamometer tests are performed at a constant low (<180 deg/s) angular velocity, which is largely different from hip flexion movements in sprinting (>850 deg/s) (Nagahara et al., 2017). Accordingly, an isokinetic dynamometer might not be suitable to measure a sprint specific hip flexion strength-power capabilities. Alternatively, Nagahara et al. (2020) recently proposed and validated an inertial measurement unit (IMU)-based hip flexion strength-power test. This proposed test consists of five repeated hip extension and flexion motions and measures hip flexion mean joint torque, angular impulse, joint work and mean power with one IMU. Given its simplicity, this test can be used as a practical field test to measure sprint specific hip flexion strength-power capabilities. Nagahara et al. (2020) revealed that the test outputs are useful to predict sprint performance, however, employed athletes from several sports with large differences in sprint performance (mean running speed for a 50-m distance being 6.5–8.5 m/s). Thus, it is still unknown whether the new hip flexion strength-power test can distinguish between faster and slower athletes within sprinters' cohort.

Step length (SL) and frequency (SF) are sub-components of running speed (Hunter et al., 2004; Nagahara et al., 2014b), and sprinters and coaches try to develop running speed through increases in SL and/or SF during their training. Thus, examining the relationship of SL and SF with the IMU-based hip flexion strength-power test variables could provide a practically important indicator of SL and/or SF in sprinting, and deepen the understanding of the hip flexion strength-power function during sprinting. Moreover, a previous study revealed that the requirements of leg extension strength-power quality changed with increasing running speed during the acceleration phase of sprinting (Nagahara et al., 2014a), suggesting that there could be alterations of association between sprint performance and the hip flexion strength-power test variables during sprint acceleration. Investigating the association of sprint performance with the hip flexion strength-power test variables in multiple sections of the entire sprint acceleration phase will lead to practically useful knowledge for the usage of the IMU-based hip flexion strength-power test.

The purpose of this study was to examine whether a recently developed IMU-based hip flexion strength-power test could be an indicator of sprint performance, SL and SF during the acceleration and maximal speed phases using sprinters.

## Method

### Participants

Sixteen well-trained male sprinters (age, 20.2 ± 1.4 yrs; stature, 1.75 ± 0.06 m; body mass, 66.9 ± 4.0 kg; personal best time of 100-m race, 11.32 ± 0.41 s ranging from 10.48 to 11.88 s) were recruited for this study. The Research Ethics Committee of the institute approved the current study, and all participants provided written informed consent prior to participating.

### Experiments

The participants sprinted 60-m twice with maximal effort. Starting blocks and the crouched start position were used. The rest period between the trials was at least 10 min. A long force platform system (TF-90100, TF-3055, TF-32120, Tec Gihan, Uji, Japan) operating at 1,000 Hz was used to record serial ground reaction force (GRF) during sprinting from the start to the 50-m mark (Colyer et al., 2018; Nagahara et al., 2018a,b).

After the familiarization trials of the hip flexion strength-power test, the participants performed in total 12 maximal effort hip flexion strength-power test trials (Supplementary Figure 1). The test was performed twice with each 1.5 and 0.75 kg ankle weight and without the weight for each leg. The number (six) of trials for each leg was selected so that the participants could perform all the trials with maximal effort without fatigue. The ankle weight was attached above the ankle joint as the center of the ankle weight was located ~0.075 m from the ankle joint center in the direction toward the hip joint center. There were reasons why the ankle weight was used. Attaching the weight near the ankle could provide moment of inertia around the hip flexion-extension axis with a relatively light load as the distance between the hip joint center and added weight was long. Moreover, the distal thigh was covered by knee brace (explained below) and the diameter around the thigh was much larger than that around the ankle. This made it difficult to attach the weight on the distal thigh. In addition, when the weight is attached around the thigh, it can provide large moment of inertia around the long axis (from hip to knee) of the thigh, which possibly causes unnecessary rotational torque around the long axis during the hip flexion trial. The hip flexion strength-power test composed of five serial hip flexion-extension motions. A specific platform and two straps were used to fix participant's torso during the trial. The participant lay on his back with the right or left leg below the hip being free to move [see Nagahara et al. (2020) for detail]. The instructions to perform the test were to flex and extend the hip joint as fast as possible without any changes in joint angles for knee (180 deg) and ankle (90 deg). The range of hip flexion and extension during the test was from hyperextension to a 20 deg flexed position from the horizontal line. The magnitude of hyperextension at the extended position was selected by the participants, as it allowed the participant to forcefully produce the flexion torque. One experimenter visually checked the range of hip extension and flexion. The participant was requested to perform the trial again when the hip extension and/or flexion ranges visually deviated. The knee (Kneebrace-Short, Alcare, Tokyo, Japan) and ankle braces (The one ankle brace, Mueller Japan, Kanagawa, Japan) were used to restrict the joint movements. The thigh motion during the test was measured using one IMU (16G, 1,500 deg/s, 200 Hz; DSP wireless 9-axes motion sensor, Sports Sensing, Fukuoka, Japan). The IMU was affixed to the lateral lower part of the thigh for each leg [see Nagahara et al. (2020) for detail]. To estimate the endpoint coordinates of leg segments during the trial, lengths for thigh, shank and foot, as well as lateral malleolus height were measured. In addition, the length from the posterior side of calcaneus to lateral malleolus in the long axis was recorded.

### Data Processing

In accordance with previous studies (Nagahara et al., 2018a,b, 2019), step-to-step running speed, SL and SF for the 50-m distance were computed from the obtained GRF data. Based on the mean running speed over the measured distance, the fastest trial for each participant was selected and used for further analysis. Moreover, averages of variables for every four steps from the 1st to 20th step and for steps from the 21st to 22nd were obtained to cancel noise possibly caused by bilateral differences and variability in cyclic movement, in reference to previous studies (Nagahara et al., 2017, 2020). Because the minimum number of steps taken for 50 m among the participants was 22, we used data for 22 steps to standardized the step number for all the participants, although the group average distance at the 22nd step was shorter than 50 m. This procedure provided six values during sprint acceleration and maximal speed phase (1st−4th, 5th−8th, 9th−12th, 13th−16th, 17th−20th, and 21st−22nd step sections).

In accordance with a previous study (Nagahara et al., 2020), a simple 2D coordinate transformation in the sagittal plane using the Euler angles recorded with the IMU was performed to calculate changes in the endpoint coordinates during the hip flexion strength-power test trial. An inverse dynamics analysis was performed to compute hip joint torque. In the case of adding the ankle weight, the joint torque was calculated using the inverse dynamics analysis with a parallel axis theorem. The location of the center of mass of the added weight was set at the 0.075 m from the ankle joint center in the direction toward the hip joint center. The body segment parameters were used to estimate the location of center of mass and the inertia parameter for each segment (Ae, 1996). The joint torque was multiplied by joint angular velocity to obtain the joint power. Based on a previous validation study (Nagahara et al., 2020), angular impulse, mean torque, positive work and mean power (concentric, accelerating hip flexion velocity by hip flexion torque) which could be accurately and validly obtained were adopted in this study. For each trial, each variable was calculated from four repetitions (repetitions two to five), then the mean value of four repetitions was computed. All the hip flexion strength-power test variables for each participant were normalized to body mass. All the variables were expressed as positive values. The values of hip flexion strength-power test from the trial with larger mean positive power were used for the statistical analyses.

### Statistical Analyses

Means and standard deviations were calculated as descriptive data. Pearson's product moment correlation coefficients were calculated to test the relationships between the variables of the hip flexion strength-power test and sprint test variables, for every step section and the total (all step sections grouped). The level of significance was set at *p* < 0.05. Threshold values of 0.1 (small), 0.3 (moderate), 0.5 (large), 0.7 (very large), and 0.9 (extremely large) were used for the interpretation of correlation coefficient as an effect size (Hopkins et al., 2009).

## Results

Running speed and SL increased until the 21st−22nd step section, while SF increased to the 9th−12th step section and decreased slightly thereafter (Table 1). All hip flexion strength-power test variables showed increases in values with increases in ankle weight (Table 2).

**Table 1:** Average distance, running speed, step length, and frequency over 50-m and for six sections of steps during 50-m sprinting.

**Variables [units]**	**Average**	**1–4**	**5–8**	**9–12**	**13–16**	**17–20**	**21–22**
Distance [m]		3.0 ± 0.3	9.0 ± 0.7	16.3 ± 1.2	24.2 ± 1.6	32.5 ± 2.1	38.8 ± 2.4
Running speed [m/s]	8.34 ± 0.21	5.56 ± 0.22	7.55 ± 0.19	8.56 ± 0.24	9.08 ± 0.24	9.34 ± 0.30	9.44 ± 0.34
Step length [m]	1.88 ± 0.10	1.30 ± 0.11	1.70 ± 0.12	1.91 ± 0.12	2.03 ± 0.12	2.09 ± 0.11	2.11 ± 0.12
Step frequency [Hz]	4.44 ± 0.25	4.29 ± 0.28	4.46 ± 0.24	4.50 ± 0.26	4.48 ± 0.26	4.48 ± 0.26	4.49 ± 0.29

*1–4, 1st to 4th step section; 5–8, 5th to 8th step section; 9–12, 9th to 12th step section; 13–16, 13th to 16th step section; 17–20, 17th to 20th step section; 21–22, 21st to 22nd step section*.

**Table 2:** Values of the hip flexion strength-power test variables in three weight conditions for the right and left hips and mean of both hips.

**Conditions**	**Variables [units]**	**Unweighted**	**0.75 kg weighted**	**1.5 kg weighted**
Right	Hip flexion angular impulse [Nms/kg]	0.478 ± 0.069	0.539 ± 0.088	0.626 ± 0.091
	Hip flexion mean torque [Nm/kg]	1.66 ± 0.30	1.82 ± 0.29	2.02 ± 0.31
	Hip flex positive work [J/kg]	0.796 ± 0.242	0.892 ± 0.336	1.047 ±.395
	Hip flex positive mean power [W/kg]	5.26 ± 1.48	5.80 ± 2.01	6.26 ± 2.32
Left	Hip flexion angular impulse [Nms/kg]	0.472 ± 0.079	0.528 ± 0.082	0.591 ± 0.070
	Hip flexion mean torque [Nm/kg]	1.65 ± 0.28	1.75 ± 0.30	1.95 ± 0.32
	Hip flex positive work [J/kg]	0.720 ± 0.186	0.800 ± 0.193	0.880 ± 0.190
	Hip flex positive mean power [W/kg]	4.69 ± 1.14	4.87 ± 1.31	5.35 ± 1.29
Mean	Hip flexion angular impulse [Nms/kg]	0.475 ± 0.059	0.534 ± 0.069	0.609 ± 0.071
	Hip flexion mean torque [Nm/kg]	1.65 ± 0.26	1.78 ± 0.25	1.98 ± 0.27
	Hip flex positive work [J/kg]	0.758 ± 0.183	0.846 ± 0.208	0.964 ± 0.259
	Hip flex positive mean power [W/kg]	4.97 ± 1.22	5.33 ± 1.27	5.81 ± 1.61

**Figure 1:**
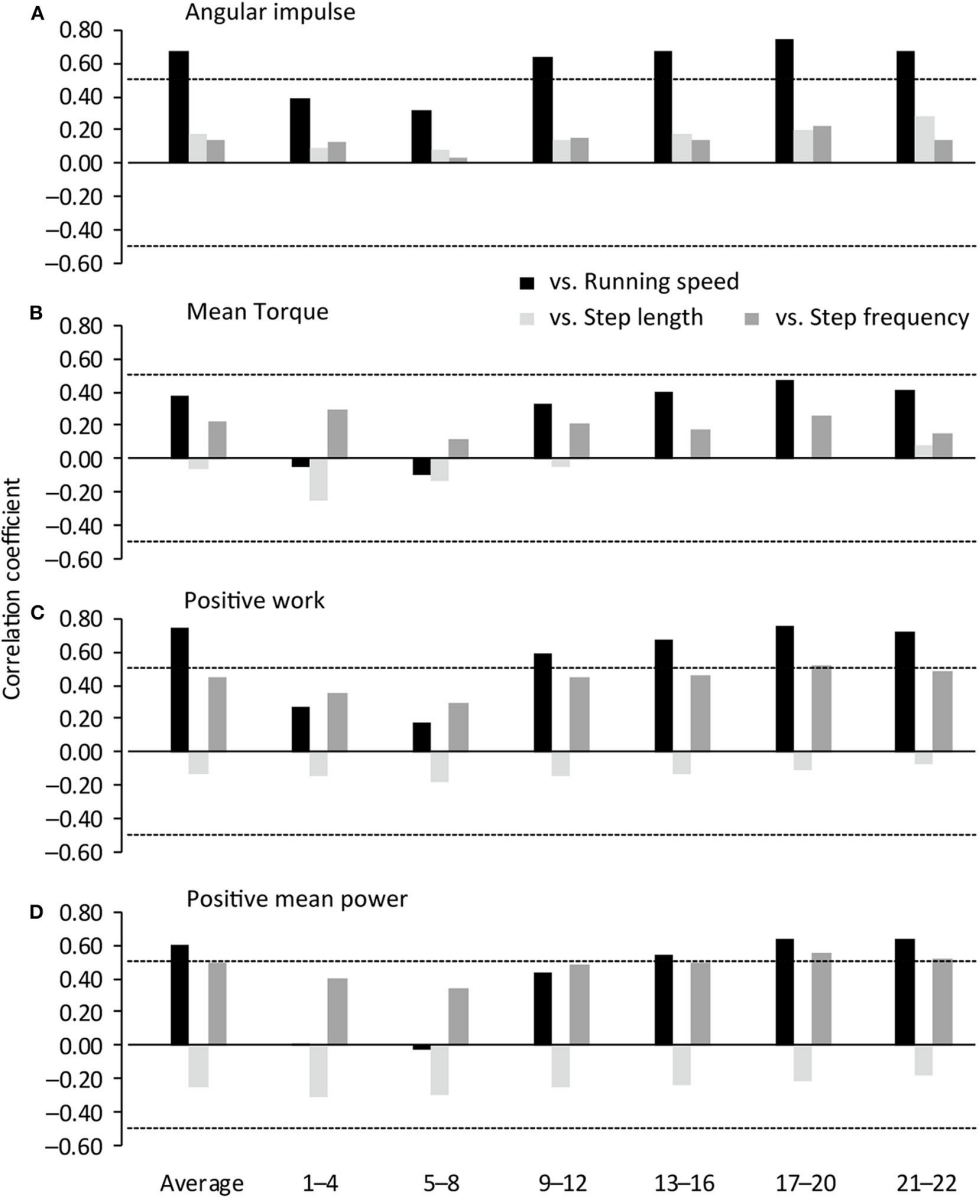
Correlation coefficients of spatiotemporal variables with IMU-based hip flexion strength-power test variables in the right leg 1.5 kg weighted condition. Horizontal dotted lines indicate *P* = 0.05. 1–4, 1st−4th step section; 5–8, 5th−8th step section; 9–12, 9th−12th step section; 13–16, 13th−16th step section; 17–20, 17th−20th step section; 21–22, 21st−22nd step section.

**Figure 2:**
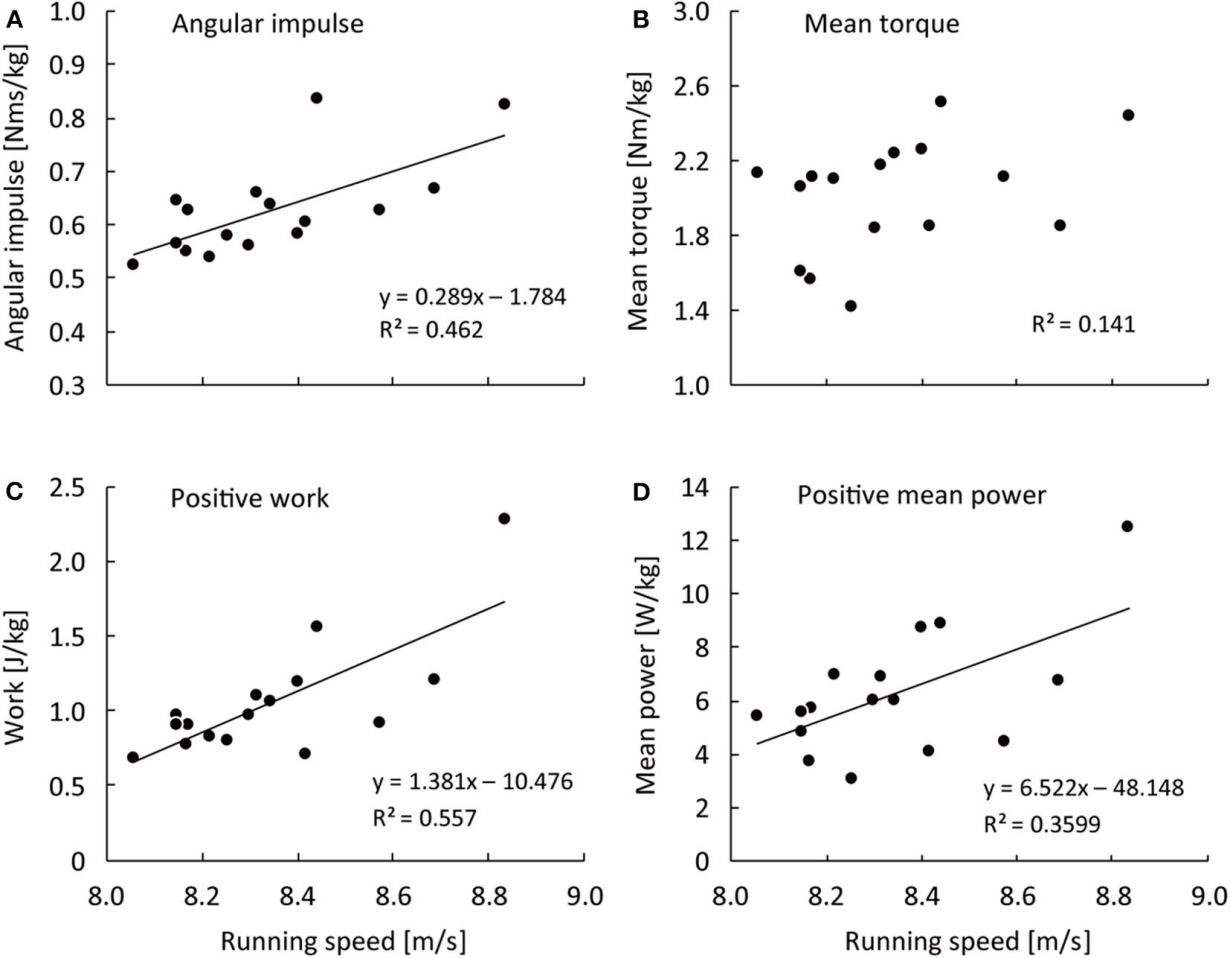
Relationship of average running speed over 50-m with angular impulse **(A)**, mean moment **(B)**, positive work **(C)**, and positive mean power **(D)** of the right hip flexion strength-power test in the 1.5 kg weighted condition.

Figure 1 shows correlation coefficients of the hip flexion strength-power test variables in the right hip 1.5 kg weighted condition with spatiotemporal variables in each steps section of sprinting. The right hip flexion angular impulse in the 1.5 kg weighted condition was positively correlated with running speed from the 9th−12th to 21st−22nd step sections and in the entire sprint (*r* = 0.637–0.746, large–very large effect) (Figures 1A, 2A). The right hip flexion positive work in the 1.5 kg weighted condition was positively correlated with running speed from the 9th−12th to 21st−22nd step sections and in the entire sprint (*r* = 0.588–0.761, large–very large effect) and with SF at the 17th−20th step section (*r* = 0.526, large effect) (Figures 1C, 2C). The right hip flexion positive mean power in the 1.5 kg weighted condition was positively correlated with running speed from the 13th−16th to 21st−22nd step section and in the entire sprint (*r* = 0.547–0.638, large effect) and with SF from the 13th−16th to 21st−22nd step section (*r* = 0.501–0.553, large effect) (Figures 1D, 2D). The right hip flexion mean torque in the 1.5 kg weighted condition was not correlated with any spatiotemporal variables (Figures 1B, 2B).

In the right hip 0.75 kg weighted condition, hip flexion positive work and positive mean power were positively correlated with running speed at the 21st−22nd step section and in the entire sprint (*r* = 0.509–0.519, large effect), and hip flexion positive mean power was positively correlated with SF at the 17th−20th step section (*r* = 0.510, large effect). In the right hip unweighted condition, the hip flexion angular impulse was positively correlated with SL at the 21st−22nd step section (*r* = 0.572, large effect), and hip flexion mean torque was positively correlated with SF at the 21st−22nd step section (*r* = 0.499, moderate effect).

In the left hip conditions, there was no significant correlation. In the 1.5 kg weighted condition with the average of two legs, running speed was positively correlated with hip flexion angular impulse at the 17th−20th and 21st−22nd step sections (*r* = 0.539–0.571, large effect), with positive work at the 9th−12th to 21st−22nd step sections and in the entire sprint (*r* = 0.515–0.690, large effect), and with positive mean power at the 21st−22nd step section (*r* = 0.519, large effect). In the 0.75 kg weighted condition with the average of two legs, running speed was positively correlated with hip flexion positive work at the 21st−22nd step section (*r* = 0.498, moderate effect). In the unweighted conditions with the average of two legs, there was no significant correlation.

## Discussion

This study firstly examined the association of the IMU-based hip flexion strength-power test variables with spatiotemporal variables in sprinting with well-trained sprinters. Because the significant correlations were mainly found in the right leg 1.5 kg weighted condition, hereafter only the results in the right leg 1.5 kg weighted condition are discussed. The main findings were 1) the 1.5 kg weighted right hip flexion strength-power test variables were associated with running speed during the later sections of sprint acceleration and maximal speed phase, and 2) the corresponding variables were associated with the SF during the later sections of sprint acceleration and maximal speed phase.

The current results demonstrate that the IMU-based hip flexion strength-power test variables, specifically angular impulse, positive work and mean power, could be an indicator of sprint performance for well-trained sprinters. Moreover, the results indicate that the greater hip flexion strength-power capabilities represented by the aforementioned hip flexion strength-power test variables are advantageous for better sprint performance during the later acceleration section approaching maximal speed and for the maximal speed phase. The importance of angular impulse, positive work and mean power derived from the IMU-based hip flexion strength-power test for sprint performance is supported by the association between the hip flexion test variables and GRF variables during sprinting, as greater angular impulse, positive work and mean power derived from the 1.5 kg weighted right hip flexion strength-power test were respectively associated with greater anteroposterior net impulse (*r* = 0.528, large effect), horizontal work (*r* = 0.506–0.653, large effect), and horizontal mean power (*r* = 0.537–0.688, large effect) during sprinting (Supplementary Table 1, Supplementary Figure 2). Among the variables, hip flexion positive work is the most useful variable to measure sprint specific hip flexion strength-power capabilities (large–very large effect). Moreover, positive work and power were the only variables which showed significant relationship with SF, underpinning the importance of these variables for achieving better sprint performance through higher SF in well-trained sprinters. In contrast to the current findings, a previous study using an isokinetic dynamometer employing well-trained sprinters did not show significant correlations between hip flexion strength-power test variables and sprint performance (Alexander, 1989). This contradiction is likely explained by differences in angular velocities and muscle contraction modalities (>600 deg/s at the peak and reactive force production in this study vs. <180 deg/s and isokinetic force production in the previous study) between the current and the previous study (Alexander, 1989). Using the same IMU-based hip flexion strength-power test, Nagahara et al. (2020) found that there were significant correlations between test variables and sprint performance with well-trained athletes (not only sprinters) with greater variation in sprint performance. As the mean running speed for a 50-m distance ranged from 6.5 to 8.5 m/s in the previous study and from 8.06 to 8.84 m/s in the present study, these previous and current findings can be integrated to demonstrate that the IMU-based hip flexion strength-power test is useful to evaluate the sprint specific hip flexion strength-power capabilities for wider and narrower ranges of difference in sprint performance.

Nagahara et al. (2017) elucidated that greater hip flexion work and mean power during the swing phase of sprinting are determinants of greater sprint acceleration performance. Thus, the requirement of greater hip flexion strength-power capabilities for greater sprint performance through larger applied propulsive force at high speed during sprinting would cause the association of the IMU-based hip flexion strength-power test variables with sprint performance, as well as GRF variables during sprinting, in the current study. With increasing constant running speed from 7 m/s to the maximal speed (9 m/s), rapid increase in SF is accompanied with acute increases in hip flexion torque, positive work and power during the initial swing phase of sprinting (Schache et al., 2011). Moreover, the greater acceleration is associated with greater increments of SF from the 16th step during sprint acceleration (Nagahara et al., 2014b). Taking these into account, the importance of hip flexion strength-power capabilities on increases in running speed through increases in SF during sprinting in the later acceleration section would lead to the significant correlations of the positive work and mean power measured using the hip flexion strength-power test with running speed and SF from the 13th−16th step section and thereafter in this study.

The significant correlations between the hip flexion strength-power test variables and sprint performance were mainly found in the right leg condition, and no significant correlation was found in the left leg condition. Although it is difficult to explain this difference between the legs, it is possible that the difference results from the dominant leg of the participants, due to the right leg being the dominant leg for all the participants in this study. In addition to the bilateral difference, the significant correlations between the hip flexion strength-power test variables and sprint performance were mainly found in the 1.5 kg weighted condition. This may be because the 1.5 kg weighted condition induced the greatest force and power productions of hip flexion (Table 2). The fact that the significant correlations were mainly shown in the right leg 1.5 kg weighted condition suggests that the hip flexion strength-power test is most useful when the dominant leg in a 1.5 kg weighted condition is employed, and this makes it possible to save time. Although the significant correlations were found between the strength-power test variables and SL in a previous study (Nagahara et al., 2020), those were found between the strength-power test variables and SF in the current study. This contradiction would be due to the range difference in sprint performance levels, as the SL is decisive for greater difference in performance levels while SF can be a determinant of small difference in performance levels (Ito et al., 2008; Salo et al., 2011). While there was no significant correlation of the IMU-based hip flexion mean torque in the right leg 1.5 kg weighted condition with sprint performance, significant correlations of the IMU-based hip flexion angular impulse in the right leg 1.5 kg weighted condition with sprint performance were found. This would be because of the difference in duration and range of hip flexion during the test as the ranges were individually different. Moreover, it can be considered that greater range of hip flexion in addition to magnitude of hip flexion torque in the test is decisive rather than solely magnitude of hip flexion torque for better sprint performance.

In conclusion, the current results demonstrate that, among well-trained sprinters, the positive work and mean power measured using the IMU-based hip flexion strength-power test in the dominant (right) leg 1.5 kg weighted condition can be an indicator of better sprint performance through higher step frequency during the later acceleration section approaching maximal speed. Given the simplicity, the hip flexion test used in this study could be useful for field-based fitness monitoring of sprinters. Specifically, the test is beneficial to evaluate sprint performance and SF during sprinting when approaching the maximal speed and at the maximal speed phase. Although this study was performed with well-trained sprinters, the IMU-based hip flexion strength-power test can possibly be useful for fitness monitoring of team-sports athletes.

## Data Availability Statement

The raw data supporting the conclusions of this article will be made available by the authors, without undue reservation.

## Ethics Statement

The studies involving human participants were reviewed and approved by Research Ethics Committee of the National Institute of Fitness and Sports. The patients/participants provided their written informed consent to participate in this study.

## Author Contributions

RN and MM contributed to conceiving, designing, and performing the experiment, to analyzing the data, and to drafting and revising the article. RN performed most of the data analysis and drafting the article. Both authors contributed to the article and approved the submitted version.

## Conflict of Interest

The authors declare that the research was conducted in the absence of any commercial or financial relationships that could be construed as a potential conflict of interest.
