# Supplementary material for: Inertial Measurement Unit Based Hip Flexion Strength-Power Test for Sprinters
Source: Front Sports Act Living. 2020 Oct 30;2:571523. doi: 10.3389/fspor.2020.571523 (PMC7739800; doi:10.3389/fspor.2020.571523)
Supplement: Supplementary file 1 [file Table_1.DOCX]

Suppl. 2 Average values of variables derived from ground reaction force data over 50-m and for six sections of steps during 50-m sprinting.

| Variables [units] | Average | 1–4 | 5–8 | 9–12 | 13–16 | 17–20 | 21–22 |
| --- | --- | --- | --- | --- | --- | --- | --- |
| Anteroposterior net impulse [Ns/kg] | .291 ± .021 | .827 ± .037 | .417 ± .023 | .247 ± .027 | .167 ± .021 | .115 ± .019 | .093 ± .017 |
| Anteroposterior net mean force [N/kg] | 2.24 ± 0.21 | 5.11 ± 0.32 | 3.30 ± 0.28 | 2.23 ± 0.34 | 1.61 ± 0.26 | 1.14 ± 0.24 | 0.93 ± 0.19 |
| Horizontal work [J/kg] | 1.76 ± 0.17 | 4.02 ± 0.27 | 2.87 ± 0.19 | 1.85 ± 0.25 | 1.23 ± 0.21 | 0.78 ± 0.20 | 0.58 ± 0.17 |
| Horizontal mean power [W/kg] | 14.1 ± 1.9 | 25.4 ± 2.1 | 23.0 ± 2.3 | 16.8 ± 3.1 | 12.0 ± 2.5 | 7.9 ± 2.3 | 5.9 ± 1.9 |

Anteroposterior net impulse at each step was calculated integrating the anteroposterior ground reaction force over the support duration. Anteroposterior net mean force at each step was calculated averaging the anteroposterior ground reaction force over the support duration. Horizontal work at each step was calculated integrating the anteroposterior power over the support duration. Horizontal mean power at each step was calculated averaging the anteroposterior power over the support duration.

1–4, 1st to 4th step section; 5–8, 5th to 8th step section; 9–12, 9th to 12th step section; 13–16, 13th to 16th step section; 17–20, 17th to 20th step section; 21–22, 21st to 22nd step section.
