# Supplementary figures and images for: Inertial Measurement Unit Based Hip Flexion Strength-Power Test for Sprinters
Source: Front Sports Act Living. 2020 Oct 30;2:571523. doi: 10.3389/fspor.2020.571523 (PMC7739800; doi:10.3389/fspor.2020.571523)

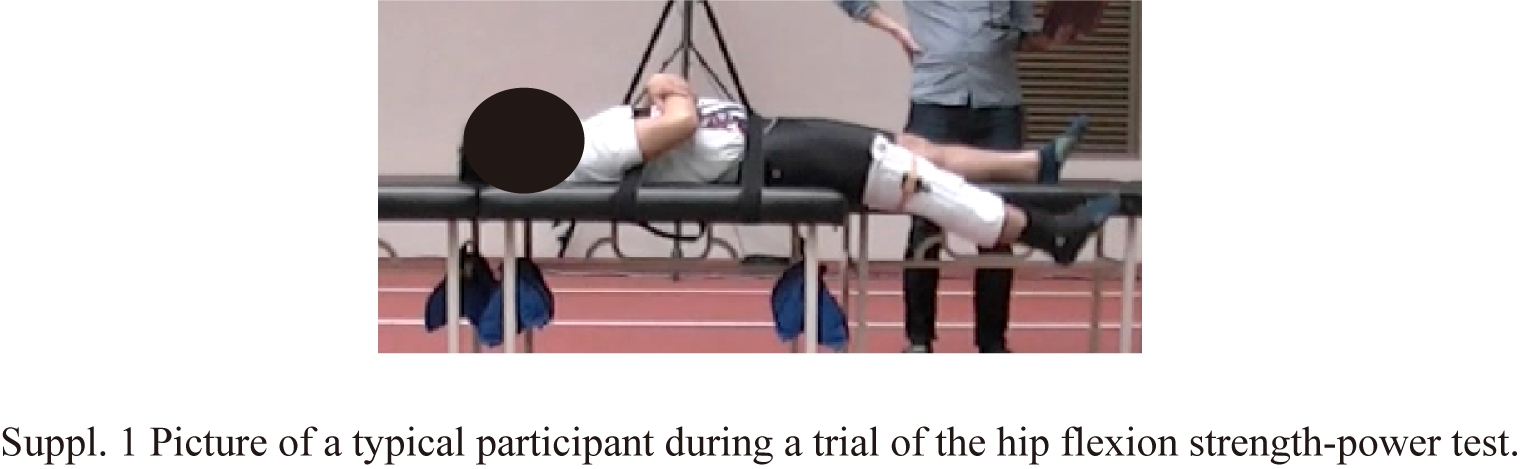

Supplement: Supplementary file 2 [file Image_1.TIF]

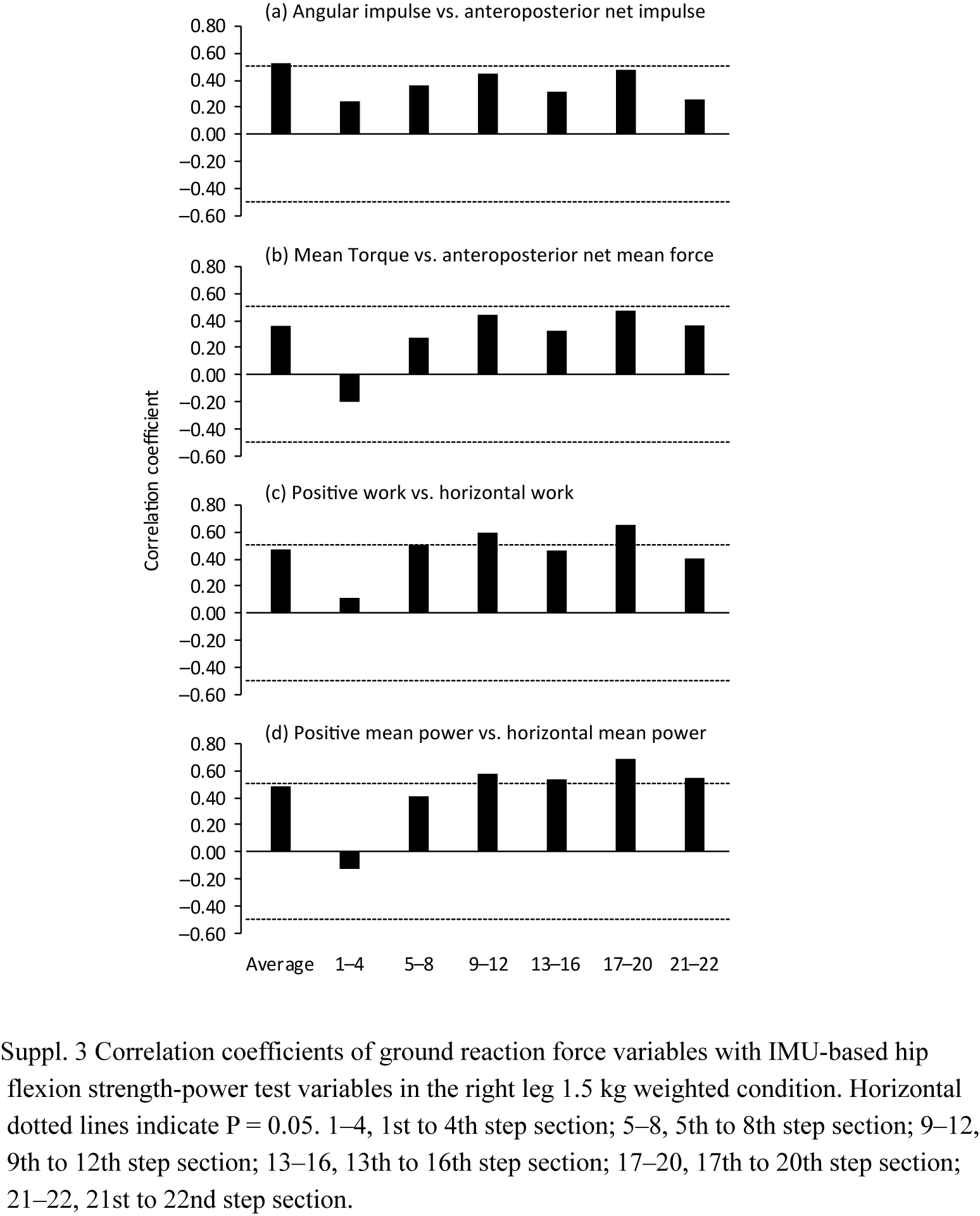

Supplement: Supplementary file 3 [file Image_2.TIF]
